# Genome Wide Analysis of the Transcriptional Profiles in Different Regions of the Developing Rice Grains

**DOI:** 10.1186/s12284-020-00421-4

**Published:** 2020-09-07

**Authors:** Ting-Ying Wu, Marlen Müller, Wilhelm Gruissem, Navreet K. Bhullar

**Affiliations:** 1 Department of Biology, Plant Biotechnology, Institute of Molecular Plant Biology, ETH Zurich, 8092 Zurich, Switzerland; 2 Present address: Temasek Life Science Laboratory, 1 Research Link, Singapore, 117604 Singapore; 3 Present address: Roche Glycart AG, Wagistrasse 10, 8952 Schlieren, Switzerland; 4 Advanced Plant Biotechnology Center, National Chung Hsing University, 145 Xingda Road, Taichung, 40227 Taiwan

**Keywords:** RNA-sequencing, Rice grain filling, Laser capture microdissection, Cross cells, Nucellar epidermis, Ovular vascular trace, Endosperm, Aleurone layer

## Abstract

**Background:**

Rice is an important food source for humans worldwide. Because of its nutritional and agricultural significance, a number of studies addressed various aspects of rice grain development and grain filling. Nevertheless, the molecular processes underlying grain filling and development, and in particular the contributions of different grain tissues to these processes, are not understood.

**Main Text:**

Using RNA-sequencing, we profiled gene expression activity in grain tissues comprised of cross cells (CC), the nucellar epidermis (NE), ovular vascular trace (OVT), endosperm (EN) and the aleurone layer (AL). These tissues were dissected using laser capture microdissection (LCM) at three distinct grain development stages. The mRNA expression datasets offer comprehensive and new insights into the gene expression patterns in different rice grain tissues and their contributions to grain development. Comparative analysis of the different tissues revealed their similar and/or unique functions, as well as the spatio-temporal regulation of common and tissue-specific genes. The expression patterns of genes encoding hormones and transporters indicate an important role of the OVT tissue in metabolite transport during grain development. Gene co-expression network prediction on OVT-specific genes identified several distinct and common development-specific transcription factors. Further analysis of enriched DNA sequence motifs proximal to OVT-specific genes revealed known and novel DNA sequence motifs relevant to rice grain development.

**Conclusion:**

Together, the dataset of gene expression in rice grain tissues is a novel and useful resource for further work to dissect the molecular and metabolic processes during rice grain development.

## Introduction

Rice (*Oryza sativa* L.) is the second most consumed crop worldwide, with nearly 3.5 billion people depending on it as a staple food. The consumption of polished rice grains is extensive in Asian countries where it provides nearly 20% of the daily caloric intake (Thomson 2003). These facts emphasize the importance of understanding the regulatory networks and molecular processes controlling grain development and grain filling in rice.

Seed development in monocots varies significantly from that in dicots. Rice grains have been anatomically characterized and comprise the embryo and specific surrounding tissues, including the endosperm, nucellar epidermis, cross-cells, ovular vascular trace and aleurone layer (Krishnan and Dayanandan 2003). The endosperm (EN) is the major storage tissue for starch that nourishes the developing embryo and contributes most significantly to human nutrition. The nucellar epidermis (NE), which differentiates from maternal tissues at around five days after flowering (DAF), supports endosperm development. The aleurone layer (AL) differentiates from the endosperm at about 8–10 DAF and has a broader metabolite profile than the central endosperm. The cross-cells (CC) produce photosynthates and transport them into the endosperm; they can be well differentiated from other tissues because of their distinct green color. The ovular vascular trace (OVT) facilitates transfer of metabolites from the flag leaf to the endosperm and to the developing embryo. The anatomical studies have provided an overview of rice grain morphology (Krishnan and Dayanandan 2003), however, the molecular processes associated with rice grain development and physiological processes are largely unknown. Here we provide a detailed analysis of gene expression in the developing rice grain tissues during the filling stage to fill this knowledge gap.

RNA sequencing (RNA-seq) has been widely used for analyzing transcriptome changes during plant development and in response to stresses. Transcriptome analysis of seeds to identify important genes and regulators expressed at different developmental stages and/or in specific tissues has also been reported. To date, the most comprehensive study on spatiotemporal gene expression networks during seed development was reported for Arabidopsis (*Arabidopsis thaliana*) by using a combination of laser-capture microdissection (LCM) and microarray analysis of tissues to identify 31 distinct sub-regions in the seed (Le et al. 2010; Belmonte et al. 2013). Among them, the chalaza is a unique tissue type highly enriched for the expression of genes related to hormone biosynthesis and ubiquitin-dependent protein catabolism. In monocots, gene expression analysis primarily focused on crop grains, including wheat, barley, maize and rice, but mostly on one or two grain sub-regions at a time (Gillies et al. 2012; Thiel et al. 2012; Gao et al. 2013; Li et al. 2014). These studies indicated complex regulatory networks in various tissue types (Lu et al. 2013b; Li et al. 2014; Zhan et al. 2015). The differential expression of genes within the maternal and filial compartments of maize kernel highlighted their significant roles in endosperm development (Zhan et al. 2015). Strong temporal specificity for expression of several genes including transcription factors were also reported during early stages of maize seed development (Yi et al. 2019). A time course analysis of transcription in barley grain revealed expression changes for genes encoding transcription factors and hormone signal transduction-related proteins, as well as genes encoding sugar-metabolism-related proteins (Bian et al. 2019). In rice seeds, embryo and endosperm regions are the most studied grain tissues for gene expression patterns (Zhu et al. 2002; Xu et al. 2012b; Xue et al. 2012; Gao et al. 2013; Ishimaru et al. 2019).

The activity and regulatory patterns of gene expression in several important sub-regions of the rice grain have not been reported. For example, the expression of genes for proteins that selectively transport micronutrients into different sub-regions during grain filling is unknown. Also, the gene regulatory networks that determine the function of the distinct tissues and cell types is poorly understood in developing rice grains. Therefore, we dissected five sub-regions of rice grains including EN, CC, NE, OVT and AL at three different time points during grain filling. Using a combination of LCM and RNA sequencing approaches, we profiled gene expression activities in the individual grain tissues. The analysis revealed the molecular mechanisms and regulatory patterns that are common or unique to these specific grain tissues. The data compendium provides an important foundation for further research, breeding and advanced biotechnological applications to improve rice grains.

## Results

### Spatio-Temporal Resolution of mRNA Profiles during Rice Grain Development

**Fig. 1:**
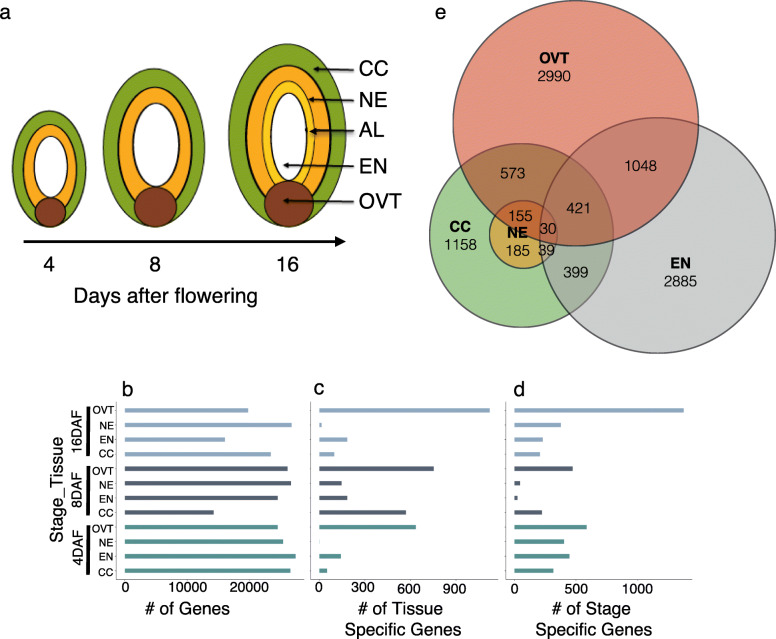
DEGs in different tissues and at developmental stages of the rice grain. **a** Graphic illustration of tissues and stages in developing rice grains collected in this study. **b** Total number of genes expressed at each developmental stage and in different tissues. Number of DEGs expressed in the specific rice grain tissues **(c)** or at the different stages of rice grain development **(d)** are indicated. **e** Venn-diagram showing the number of AL-specific DEGs compared pair-wise to CC, EN, NE and OVT DEGs at 16-DAF, respectively. NE: Nucellar Epidermis; CC: Cross Cells; OVT: Ovular Vascular Trace; EN: Endosperm; AL: Aleurone Layer

Gene expression was analyzed in five sub-regions of the developing rice grain comprising the CC, NE, OVT, EN and AL tissues that were collected by laser-capture micro dissection at 4, 8 and 16 DAF (Fig. 1a, Fig. S1). RNA sequence reads were quality checked and over 20,000 genes were identified with statistically significant expression (reads higher than 10, *p* < 0.001). Genes with expression specific to each sub-region and grain development stages were compared using hierarchical clustering and principal component analysis (PCA). The biological replicates from individual sub-regions clustered together and PCA collectively identified 79% variance in our dataset (Fig. 1, Fig. S2). The dataset was further analyzed to separate genes for their tissue- and stage-specific expression. The genes with a preferentially higher expression level (with the highest number of reads, p < 0.001) in a particular sub-region or at a particular developmental time point were assigned to be tissue-specific and stage-specific differentially expressed genes (DEGs), respectively. In total, 10,037 DEGs were identified. In order to further characterize the sub-region and stage-specific genes, gene ontology (GO) analysis was conducted on DEGs using PlantGSEA (Yi et al. 2013). Significantly overrepresented GO terms and metabolic pathways (*p* < 0.01) were used for further comparison. Selected genes were also analyzed for their expression in these tissues by quantitative PCR (qPCR), generally validating the RNA-Seq results (Fig. S3).

#### Tissue-Specific DEGs

**Fig. 2:**
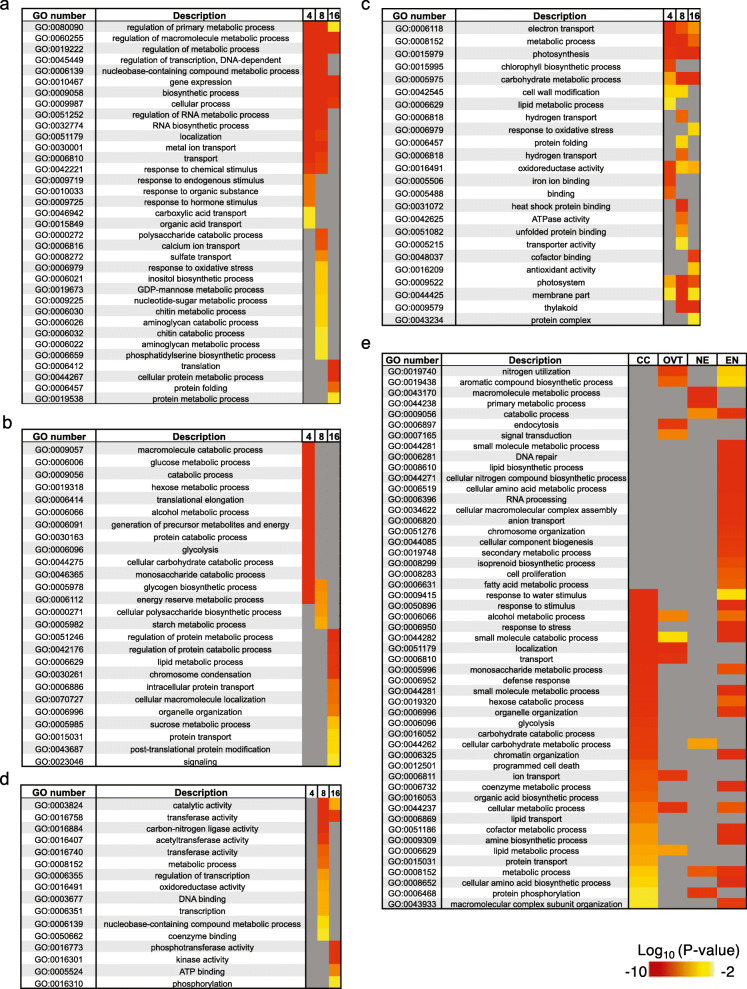
Gene Ontology (GO) analysis of the DEGs in different tissues of the developing rice grain at 4, 8 and 16 days after flowering (DAF). Significantly (*p* < 0.01) overrepresented GO terms in **(a)** OVT, **(b)** CC, **(c)** EN, **(d)** NE and **(e)** AL are shown as heatmaps. If a certain GO term was not significantly enriched (*P*-value > 0.01) in a certain tissue or stage, the cell is colored grey

In OVT, 635 and 753 DEGs were identified at 4 and 8 DAF, respectively. The most significantly enriched GO categories for these genes are *iron binding*, *transporter*, *transcriptional activity*, and *phytohormone biosynthesis and signalling*. Among the 1123 DEGs identified as OVT-specific at 16 DAF, the over-represented GO categories included *protein translation*, *ribosomal activity*, *protein folding* and *macromolecular metabolic process* (Fig. 2a, Fig. S4a and Table S1). In the EN-specific set of genes, 143, 186, and 185 DEGs were identified at 4, 8 and 16 DAF, respectively. The enriched GO terms for EN-specific genes were associated with metabolism and providing energy, such as *starch and glucose metabolism*, *glycolysis*, *transportation*, and *protein and lipid metabolism*. Starch biosynthesis and microtubule-associated genes were also highly expressed in EN at 4 and 8 DAF, suggesting that endosperm cells are still enlarging during grain filling (Fig. 2b and Table S1). In the case of NE, one DEG at 4 DAF, 147 DEGs at 8 DAF, and 16 DEGs at 16 DAF were identified. The enriched GO terms for the 8 DAF NE-specific DEGs included *transferase*, *metabolic activity*, *transcription*, and *coenzyme binding*, consistent with the role of the NE in supporting EN development (Fig. 2c). Similarly, consistent with the suggested role of CC during grain development, the overrepresented GO terms among the 53, 572, and 99 DEGs identified in the CC tissue at 4, 8 and 16 DAF, respectively, included *photosynthesis*, *transporter activity*, *metal binding*, *catalytic activity*, and *metabolic processes* (Fig. 2d and Table S1). Since AL tissue was collected only at 16 DAF, we performed pairwise comparisons of gene expression in the AL tissue with CC, EN, NE, and OVT tissues at 16 DAF. This revealed 1158, 2885, 185, and 2990 DEGs, respectively (Fig. 1e). Genes associated with macromolecule metabolism, amino acid metabolism, secondary metabolism, and lipid metabolism as well as vitamin B biosynthesis and transport were expressed in AL. These results suggest an important role of the AL in synthesis and transport of metabolites during grain filling in rice. Additionally, some stress responsive genes were also identified among the AL-specific DEGs (Fig. 2e, Fig. S4b and Table S2).

#### Stage-Specific DEGs

**Fig. 3:**
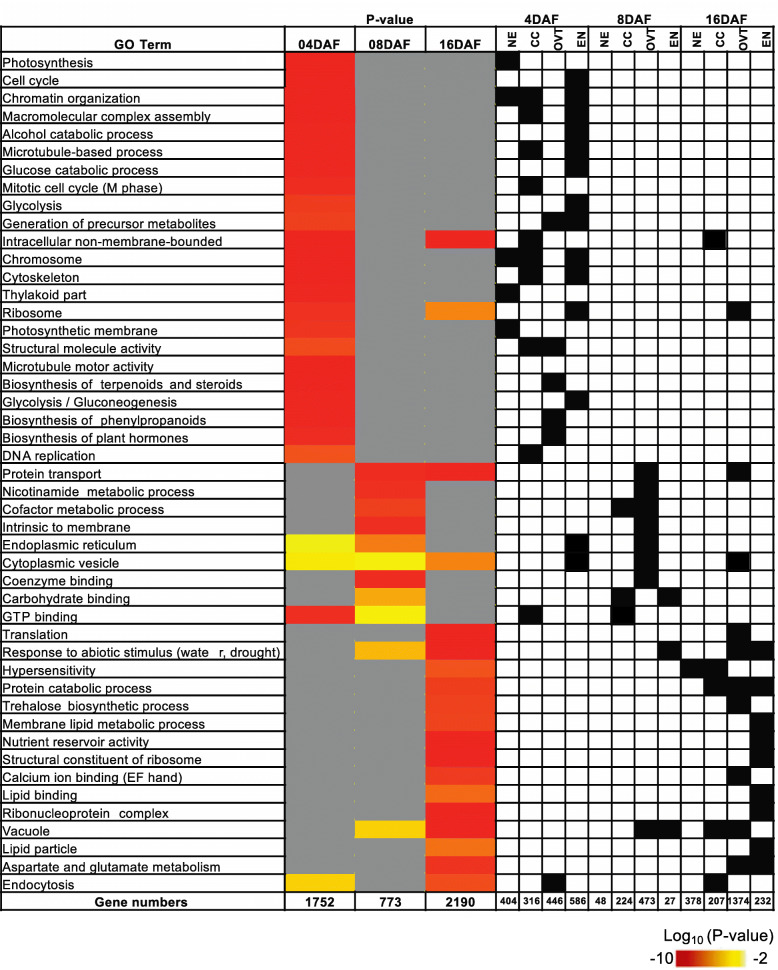
GO analysis of the DEGs in the developing rice grain at 4, 8 and 16 DAF. GO terms from *biological process* are shown. Significantly (p < 0.01) overrepresented GO terms at 4, 8 and 16 days after flowering (DAF) are shown as heatmap. Color key represents Log_10_ (P-value). Black boxes in the right panel indicate the specific tissues with the enriched GO terms. If a certain GO term was not significantly enriched (P-value > 0.01) in a certain tissue or stage, the cell is colored grey

Among the DEGs, 1752 DEGs at 4 DAF, 773 DEGs at 8 DAF, and 2190 DEGs at 16 DAF were identified as growth stage-specific genes (Fig. 1). The genes in the 4 DAF-specific set were related to *chromatin assembly*, *DNA replication*, *cell mitosis and division* in NE, EN and CC tissues, and to *hormone regulation* and *glycolysis* in OVT. The 8 DAF-specific genes were related to major *CHO metabolism* and *transporter activity* in OVT and EN. Over-represented GO categories of stage-specific gene sets indicate a transition to translation and protein biosynthesis to establish protein localization and secondary metabolism, especially in CC, OVT and EN tissues at 16 DAF (Fig. 3), which is consistent with previous studies on seed maturation (Gillies et al. 2012).

### Diverse and Complex Regulatory Networks Controlling Grain Filling in Rice

**Fig. 4:**
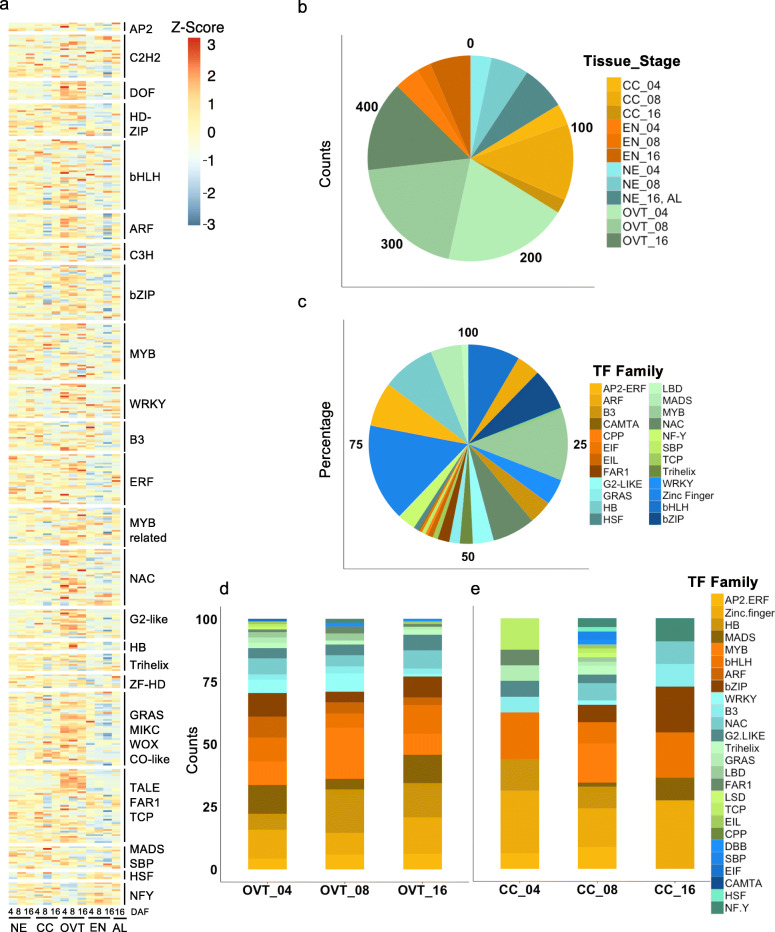
Analysis of DEGs encoding transcription factors (TFs). **a** Overview of heatmap for all differentially expressed TF encoding genes. **b** Percentage of differentially expressed TF-encoding genes in different sub-regions and at different growth stages of the developing rice grains. **c** Distribution of TF families among the differentially expressed TF encoding genes in the data set. **d** Distribution of TF families in the OVT and CC **(e)** tissues at different stages of the developing rice grain

In order to explore the role of transcription factors (TFs) in rice grain development, we surveyed about 1500 expressed genes encoding TFs in the rice genome. Of these, 470 TF genes were differentially expressed in our data set (Fig. 4a, and Table S3). The OVT tissue had the maximum number of DEGs encoding TFs, comprising 21.3%, 21.5% and 15.5% of TFs at 4, 8 and 16 DAF, respectively (Fig. 4b). Diverse types of TF-encoding genes were found to be differentially expressed, with the majority of these OVT-specific TFs belonging to bHLH and MYB families of TFs (Fig. 4c). Several of these TF family members, such as ABI3/VP1, ARF, Aux/IAA, B3, ARR and GRAS, have important roles in hormone regulation. Additionally, the TFs known for their roles in vascular development, cell growth regulation and differentiation, such as MYB, SBP, MADS, NAC, bZIP and HB family members, were also differentially expressed in the OVT. Growth stage-specific differences in TF expression profiles were also observed by enrichment analysis and co-expression network analysis. Genes encoding members of MADS and bZIP TF families showed higher expression at 4 and 8 DAF, while those encoding MYB, HB and WRKY TFs had elevated expression at 16 DAF in the OVT (Fig. 4d, Fig. S5a). The CC tissue-specific dataset revealed a higher proportion of TFs as compared to NE, EN and AL tissue-specific sets, especially at 8 DAF (13%) (Fig. 4e). The enriched TF families included WRKY, HB and Zinc finger TFs, most of which are known to be involved in seed development (Fig. 4e) (Zhang et al. 2011; Joseph et al. 2014). Chi-square test and co-expression network analysis showed that zinc-finger and AP2-ERF TF families were overrepresented in CC at 4 and 8 DAF (Table S3, Fig. S5b). In addition, nine TFs were highly expressed in EN at 16 DAF, and most of these are involved in seed maturation or germination (Kim et al. 2008; Lasserre et al. 2008). Two genes encoding AP2-like ethylene-responsive TFs (*Os04g0653600* and *Os05g0437100*) and three heat shock TFs (*Os02g0232000*, *Os06g0553100* and *Os10g0419300*) were more highly expressed in the AL tissue (Table S3). *OsWRKY71* (*Os02g0181300*) had the highest expression level in the AL tissue, as was previously reported (Zhang et al. 2004). Together, these data suggest that reprogramming events in the developing rice grains comprise a complex coordination of different TFs in each sub-region tissue and at different developmental time points.

### Expression Profiles of Genes Related to Hormone Biosynthesis and Signalling

**Fig. 5:**
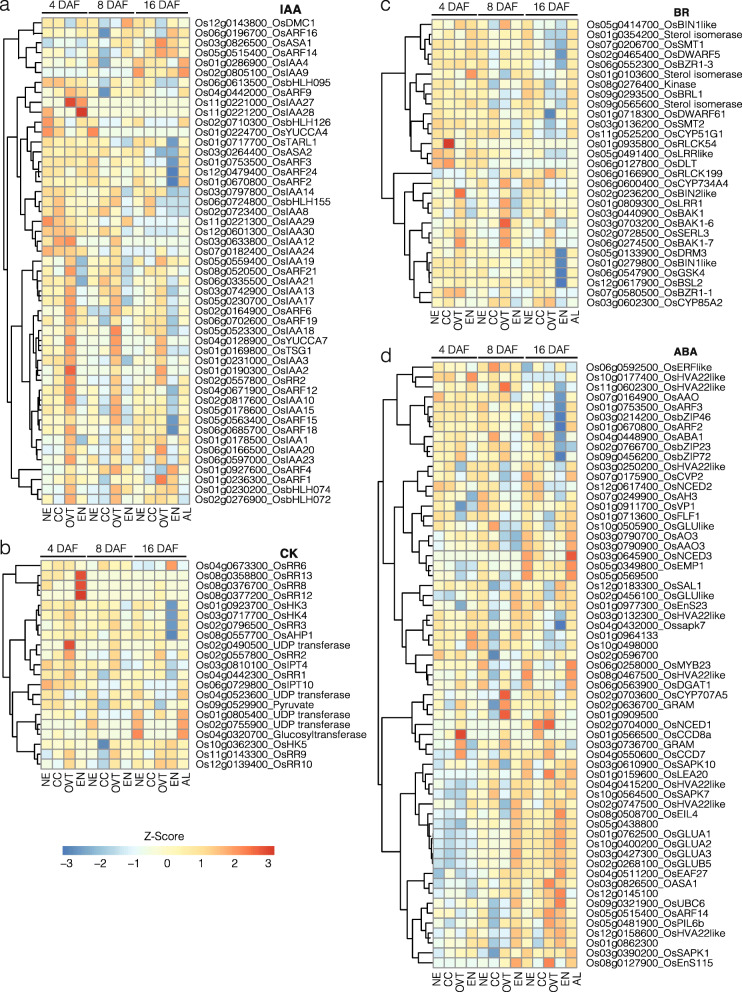
Expression profiles of DEGs related to hormone metabolism and transport in different tissues of the developing rice grain. **a** Genes related to auxin (IAA) biosynthesis, signalling and transporters. **b** Genes related to cytokinin (CK) metabolism, transporters and receptors. **c** Genes related to brassinosteroid (BR) metabolism, transporters and signalling. **d** Genes related to abscisic acid (ABA) signalling. The gene-normalized signal intensities are shown in the heat maps using Z-Scores. DAF: Days after flowering

Since plant hormones regulate grain development, we analyzed the expression of genes related to auxin (IAA), gibberellin (GA), brassinosteroid (BR), cytokine (CK), abscisic acid (ABA) and ethylene (ET) biosynthesis and metabolism. In general, BR-, CK- and GA-related genes had higher expression levels at 4 and 8 DAF in all sub-regions. The genes related to IAA biosynthesis and metabolism had relatively stable expression in OVT during 4 and 8 DAF, but exhibited slightly decreased expression at 16 DAF (Fig. 5, Fig. S6a and 6b, and Table S4).

The genes encoding IAA biosynthesis enzymes, including *OsYUCCA7*, *OsYUCCA4*, *OsASA1* and *OsASA2* (Mano and Nemoto 2012), were highly expressed in the OVT tissue at 4 and 8 DAF, and showed reduced expression at 16 DAF. Genes encoding IAA transporters and related TFs, such as *Aux/IAA*, were expressed higher in OVT at 4 and 8 DAF. IAA signalling genes, however, were highly expressed in the AL tissue as compared to the EN at 16 DAF (Fig. 5a). GA20ox is a key enzyme in GA biosynthesis (Fleet and Sun 2005) and among GA20ox encoding genes in rice, *Os01g09300* had higher expression level in OVT at 4 DAF. Notably, GA-related genes were expressed at relatively low levels in EN at every time point (Fig. S5a). CK biosynthesis-related genes including *OsRR2* and *OsRR6* (Hirano et al. 2008) were also preferentially expressed in the OVT tissue at 8 DAF (Fig. 5b). Only few BR biosynthesis-related genes were differentially expressed in our datasets. Among them, *OsDWARF* (*Os03g0602300*) encodes the BR enzyme C-6 oxidase, which is involved in seed development and panicle elongation (Mori et al. 2002); this gene was differentially expressed in CC and OVT tissues at 8 and 16 DAF. Several of the BR signalling-related genes were found to be differentially expressed, especially in the OVT tissue. *OsCYP* genes involved in BR synthesis are known to regulate seed size (Wu et al. 2008); they were expressed highly in the OVT tissue at 4 and 8 DAF (Fig. 5c). Genes encoding enzymes for ABA biosynthesis exhibited higher expression in the AL and the EN tissues, while the ABA signalling-related genes were preferentially expressed in the AL tissue at 16 DAF (Fig. 5d). This result is in a good agreement with previous reports, in which genes involved in ABA biosynthesis and signalling, including *NCED*, *AAO*, *bZIP* and *PP2C* (*HVA*), were enriched in the EN in late developmental stages (Xue et al. 2012) (Fig. 5d). The expression pattern of genes encoding ethylene (ET) signal transduction genes was quite stable during 4, 8 and 16 DAF (Fig. S6b). The expression of ethylene responsive TF genes was low in EN at 16 DAF.

### Transporters Are Preferentially Expressed in OVT

**Fig. 6:**
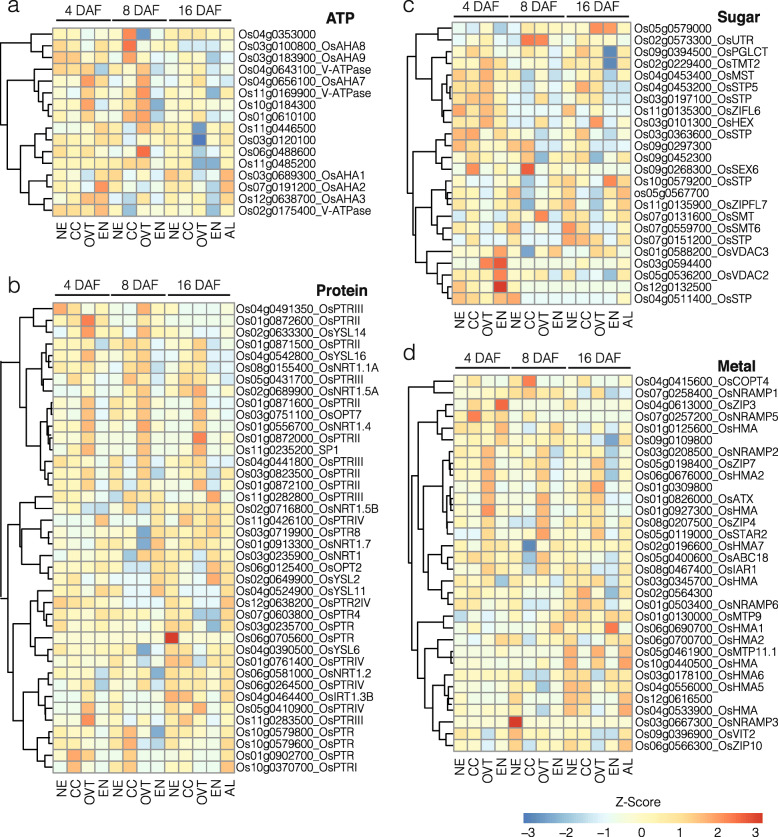
Expression profiles of DEGs related to transporters. **a** Genes involved in ATP synthesis and transport. **b** Genes involved in protein transport. **c** Genes involved in metal transport. **d** Genes involved in sugar transport. The gene-normalized signal intensities are shown in the heat maps using Z-Scores. DAF: days after flowering

In order to gain insights into nutrient transport routes in the rice grain, we extracted the list of genes encoding different transporters from MapMan (https://mapman.gabipd.org) and examined their expression patterns in our set of DEGs. Overall, the expression level of genes encoding nutrient transporters, including transport ATPases and sugar-, nitrate- and sulfate-transporters, was higher in OVT, CC, and NE at 4 and 8 DAF. The expression level of genes encoding amino acid and protein transporters was higher at 16 DAF in OVT, CC and AL. These genes in general had a low expression in EN as compared to the other tissues (Fig. 6, Fig, S6c and 6d, and Table S5).

Fifteen genes encoding P- and V-type transport ATPases showed the highest expression at 8 DAF in OVT and CC. Three of these genes expressed higher in CC at 8 DAF and among these, *Os03g0183900* is a plasma membrane H^+^-ATPase that is specifically expressed in seeds (Baxter et al. 2003) (Fig. 6a). Genes encoding protein and sugar transporters expectedly had higher expression levels at 4 and 8 DAF in EN, while their expression decreased at 16 DAF in EN but increased in AL (Fig. 6b and c). We also identified several genes for sugar transporters that were preferentially expressed in OVT at 4 and 8 DAF. Six putative sugar transporters showed higher expression at 4 DAF in the OVT (*Os02g0229400*, *Os04g0453400*, *Os04g0453200*, *Os03g0197100*, *Os11g0135300*, and *Os03g0101300* (Fig. 6c), supporting the view that OVT has a significant role in transporting sugars and other metabolites to the developing EN and embryo. Three genes encoding putative sugar transporters (*Os03g0594400*, *Os12g0132500*, and *Os04g0511400*) had their highest expression levels at 4 DAF in EN. The expression of known genes encoding sucrose transporters, including *OsSUT1*, *OsSUT2*, *OsSUT3*, *OsSUT4* and *OsSUT5* (Zhu et al. 2002), was similar at 4, 8 and 16 DAF in all sub-regions. Most of the genes encoding amino acid transporters (Zhao et al. 2012), including *OsAAP2*, *OsAAP3*, *OsAAP5*, *OsAAP6* and *OsAAP7*, were preferentially expressed at high levels in OVT but not in EN at 4, 8 and 16 DAF (Fig. S6c). However, *OsCAT11*, *OsATL6* and *OsPROT2* showed an increased expression in EN at 16 DAF (Fig. S6c). The genes encoding nitrate, phosphate and sulfate transporters were preferentially expressed in OVT at 4 and 8 DAF but preferentially expressed only later in NE, CC and AL at 16 DAF (Fig. S6d). For example, the high affinity nitrate transporter *OsNAR2.1* (Feng et al. 2011) was expressed highly in OVT at 4 DAF (Fig. S6d). *OsPHO1;2* and *OsPHO1;1*, which tightly regulate phosphate loading into xylem in rice and plant fitness (Secco et al. 2010; Jabnoune et al. 2013), were upregulated in OVT at 8 DAF and in CC at 16 DAF, respectively. *OsSULT3;3* and *OsSULT3;4*, which showed highest expression levels in AL at 16 DAF and in OVT at 8 DAF, respectively, are known to be induced by heavy metal stress (Buchner et al. 2004; Kumar et al. 2011) (Fig. S6d). *OsSULT3;5* was the only gene among the genes encoding sulfate transporters that showed the highest expression level in EN at 16 DAF (Fig. S6d).

**Fig. 7:**
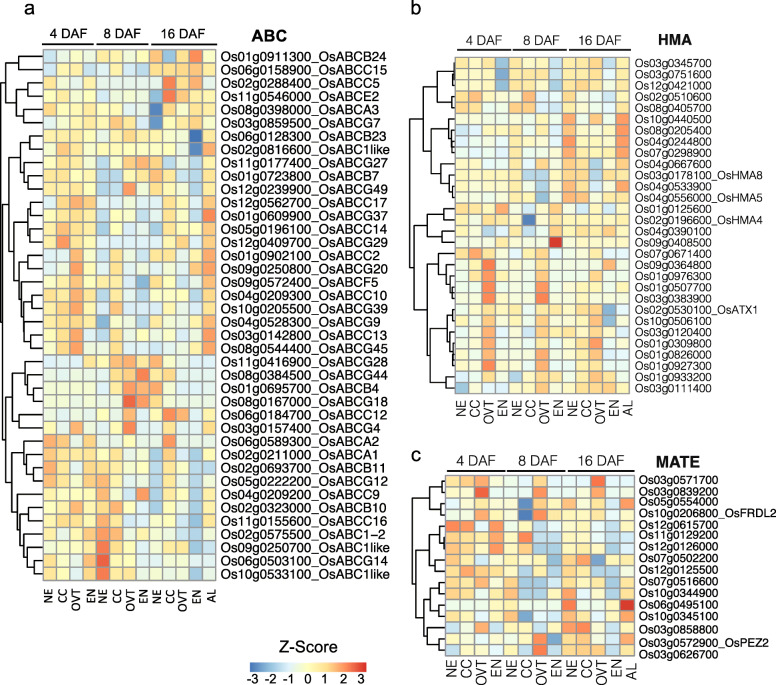
Expression profiles of DEGs related to ATP-binding cassette (ABC), heavy metal ATPase (HMA) and multidrug and toxic compound extrusion (MATE) transporter families. **a** Expression pattern of genes from the ABC transporter family. **b** Expression pattern of genes from HMA transporter family. **c** Expression pattern of genes from MATE transporter family. The gene-normalized signal intensities are shown in the heat maps using Z-Scores. DAF: days after flowering

The expression of genes encoding iron- and zinc-related transporters, such as NRAMP, ABC, HMA and ZIP (Lanquar et al. 2005; Kobayashi and Nishizawa 2012), was higher at 4 and 8 DAF, but significantly decreased at 16 DAF (Fig. 6d, Fig. 7 and Tables S5 and S6). As expected, EN had the lowest expression of genes encoding metal transporters, although they were more highly expressed in AL at 16 DAF (Fig. 6d). Expression of *NRAMP* gene family members including *OsNRAMP1*, *OsNRAMP2*, *OsNRAMP3* and *OsNRAMP5* was higher in OVT and CC but lower in EN at 4 and 8 DAF (Fig. 6d). Similarly, a group of HMA family genes showed significantly higher expression level in OVT at 4 and 8 DAF, and in NE and AL at 16 DAF (Fig. 7b). Among the ZIP family genes, *OsZIP4* and *OsZIP7* showed higher expression level in OVT at 4 and 8 DAF, while *OsZIP3* showed highest expression in EN (Fig. 6d). Genes encoding proteins for iron acquisition and transport such as *OsFRDL2* (Yokosho et al. 2016), *OsOPT*, *OsYS* and *OsCOPT3* were also expressed more highly in OVT and CC at 4 and 8 DAF (Figs. 6b and d and 7c). Together, the expression pattern of genes encoding metal transporters is consistent with the low levels of iron and zinc in the rice endosperm that is consumed as polished rice.

### Novel *cis*-Regulating Elements Associated to OVT and CC Specific Genes

Since a significant number of genes that were differentially expressed in OVT and CC encode transcription factors as indicated by GO categorization, we analyzed the DEG sequences to identify known or novel TF regulatory DNA sequence motifs in their promoter or coding regions. We searched approximately 1000 bp proximal and distal to the ATG start codon for sequence motifs and identified them using the MEME and JASPAR plant databases. Several common motifs for TF families associated with seed development were identified among the OVT- and CC-specific DEGs (Tables 1 and 2) (Papi et al. 2000; Sreenivasulu et al. 2006; Wang et al. 2010). For example, the motifs for the two MADS family TFs SOC1 and PI were found in OVT and CC DEGs at 4, 8 and 16 DAF. Similarly, the high mobility group box HMG-I/Y for the HMG TF family and the idl motif that is bound by the zinc finger TF family were also enriched in our dataset during all three development stages. Several motifs were enriched only in the OVT specific DEGs. Of note, ABI3 (GCATG), a binding site of ABI/VP3 transcription factor related to auxin signaling, and BES1 (CACGTG) motif, related to BR signalling were enriched only in OVT-specific genes at 8DAF, suggesting an important role of phytohormone related transcription factors in early grain filling. At 16 DAF, AtMYB84 (GGTnGGT) and AtSPL8 (GTAC) were enriched motifs in OVT but no motifs specific to CC were detected as enriched at this development stage (Table 1). We also identified a novel motif “GGAGGA” in OVT and CC specific genes at 8 DAF. From the analysis using GO-MEME (Buske et al. 2010) and cross comparison with other plant species, it is likely that this motif is involved in transporter and hormone regulation. Another motif “GCCGCC” was identified as unique, but a high GC content motif is suggested to be commonly found in rice gene promoters (Lenka et al. 2009, 2011). In the distal regions of OVT and CC specific genes, we found motif “GCATGC” as enriched in OVT specific genes at 4 and 8 DAF, and motif” GAGAGA” enriched in CC specific genes at 8 DAF (Table 2). We compared these two motifs with known motifs using TOMTOM (Gupta et al. 2007) software. “GCATGC” was similar to BR signalling-associated regulatory element BES1 and “CCTCC” was similar to MYB regulatory elements (Table 2). 
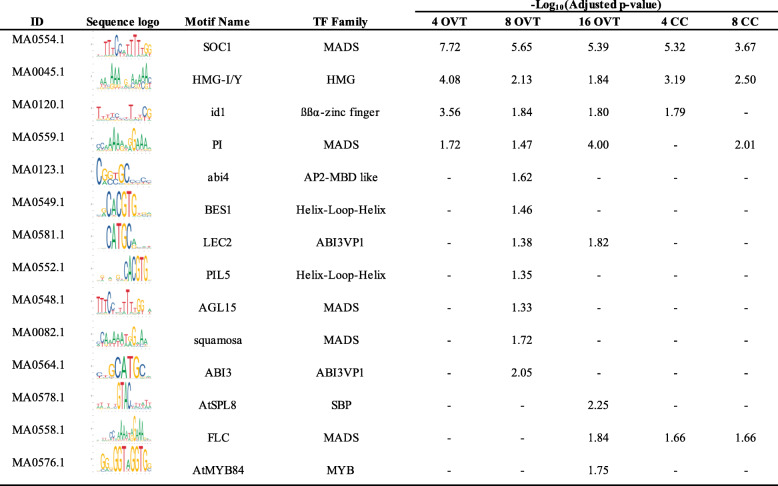

**Table 1:**
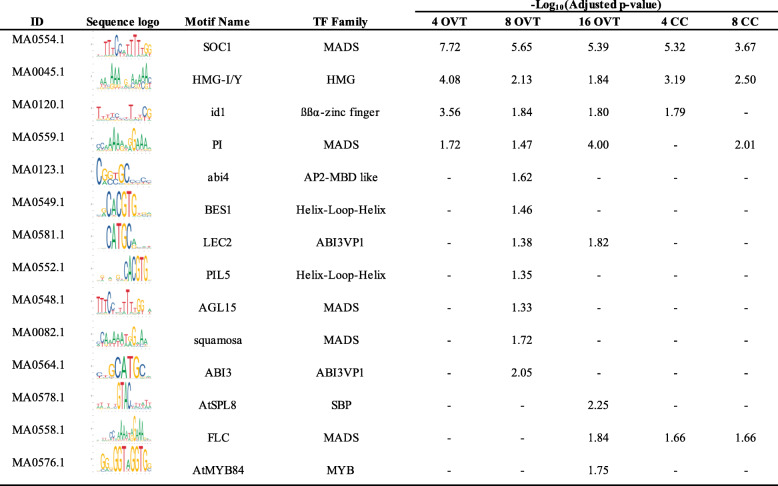
Summary of overrepresented cis-regulatory DNA sequence motifs identified from MEME analysis


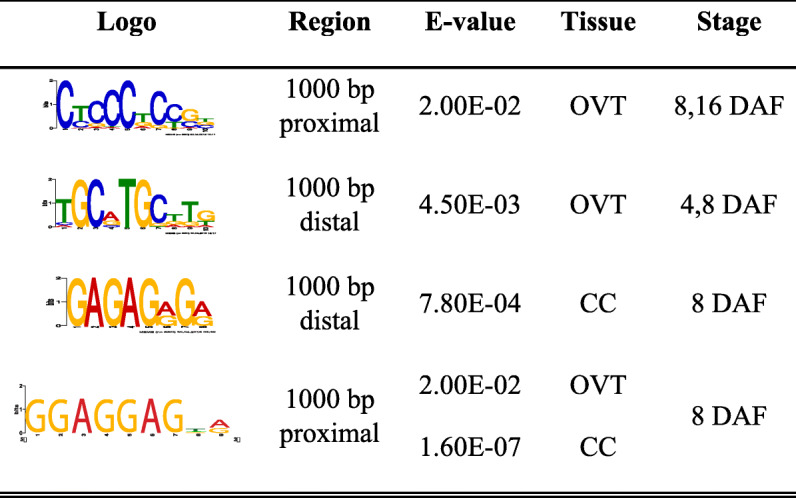

**Table 2:**
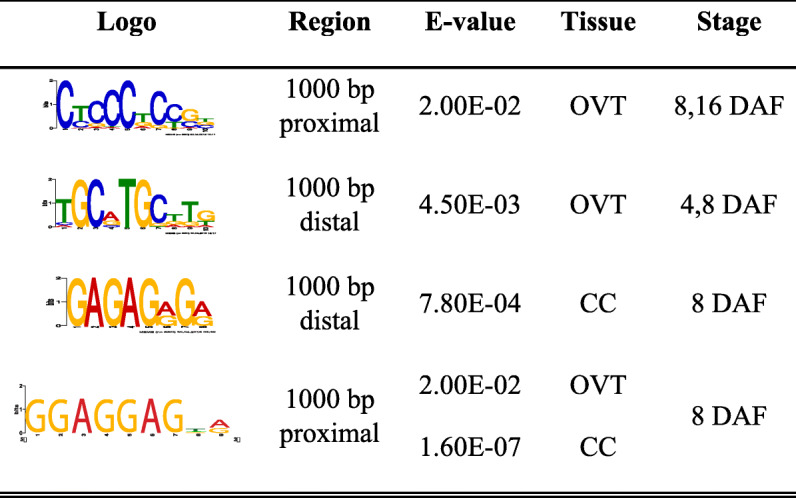
Summary of novel overrepresented cis-regulatory DNA sequence motifs identified from MEME analysis

## Discussion

**Fig. 8:**
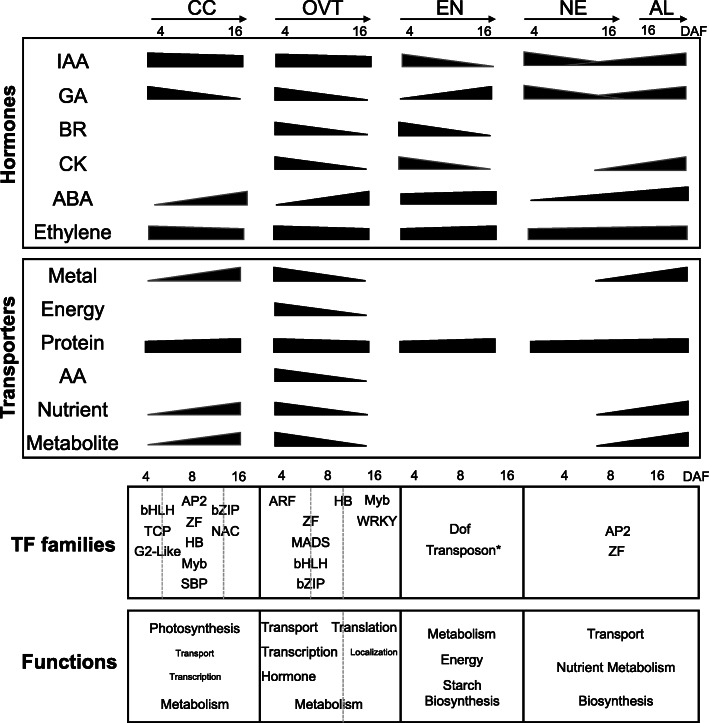
Summary of spatio-temporal gene expression regulation in different tissues during rice grain development. Upper panel: Changes in the expression of hormone-regulated genes and genes encoding transporters in CC, OVT, EN, NE and AL are summarized. Enriched TF families in different sub-regions during different developmental stages are indicated. The sloped bars indicate the activity of hormone or transporter genes in the respective stage and tissues. Lower panels: DEGs of TFs enriched in the five tissues at different stages of grain development (4, 8 and 16 DAF; separated by dashed lines). TFs that were enriched in more than one stage are shown across two columns. The enriched metabolic, physiological and cellular functions in each tissue are each stage are shown. Functions that were enriched at more than one stage are shown across the column. The different font size of the terms indicate their degree of enrichment

Seeds store the embryo and genetic information for the next plant life cycle, and provide nearly 70% of the human caloric intake worldwide. Therefore, understanding gene expression in different seed tissues will help to identify important regulators of seed development and grain filling. Several transcriptome studies have characterized gene expression in different sub-regions of seeds from various grain crops (Thiel et al. 2012; Pfeifer et al. 2014; Zhan et al. 2015). Earlier studies of rice seeds focused only on transcription in the embryo and endosperm (Xu et al. 2012b; Gao et al. 2013), but gene expression patterns in different tissues of the developing grain are still unknown in rice and other grain crops. Our comprehensive analysis of developing rice seed tissues revealed the expression of several tissue- and stage-specific genes encoding proteins involved in transcriptional and hormone regulation as well as transport processes. Figure 8 summarizes the results and captures the complexity of tissue-specific gene expression during rice grain development.

The GO analysis revealed common and unique stage- and tissue-specific genes, indicating that the different tissues share certain molecular and metabolic processes but also have distinct functions during rice seed development, which is consistent with earlier morphological and molecular studies (Krishnan et al. 2001; Zhu et al. 2002; Krishnan and Dayanandan 2003; Xu et al. 2012b; Xue et al. 2012). Endosperm transfer cells (ETC) in barley and wheat differ from aleurone cells and are thought to transfer hormones, amino acids, sugars, micronutrients, other solutes and water to the endosperm (Offler et al. 2003; Thiel et al. 2012), which are important for grain filling and quality (Jia et al. 2011; Sun et al. 2012; Xu et al. 2012a). Most of the genes encoding divalent metal transporters, including *HMA*, *ZIP*, *YSL*, *NRAMP*, *MTP*, and *VIT* families, respond to alterations of zinc and iron concentrations in ETC of barley seeds (Tauris et al. 2009). Auxin, glucose, ethylene and reactive oxygen species initiate signal cascades leading to the development of ETC (Thiel et al. 2012). It has been suggested that the rice OVT has similar functions as the ETC (Krishnan and Dayanandan 2003; Li et al. 2008). In this case, minerals and nutrients transported to the developing rice seed are unloaded from the OVT, which contains both phloem and xylem cells, into the aleurone and endosperm during grain filling (Lu et al. 2013a; Zhao et al. 2014). Our transcriptome data support this suggestion because genes encoding proteins involved in hormone and different transport functions are overrepresented among the genes expressed in OVT at 4 and 8 DAF during seed development. For example, *NRAMP*, *HMA*, and *ZIP* family genes as well as genes encoding proteins related to auxin and ethylene biosynthesis and signalling were preferentially expressed in OVT at 4 and 8 DAF.

Although the EN and AL tissues share several metabolic pathways, our data also reveals their distinct biochemical functions. Genes specific to AL are involved in secondary metabolism, lipid metabolism, and protein processing, while the EN-specific genes, as expected, are involved in starch synthesis, sugar and energy metabolism. At 16 DAF, the transcription of most genes in the EN was reduced compared to the AL, suggesting an important function of AL in providing metabolites and energy to the developing grains at this stage. Consistent with our results for EN specific genes, an earlier transcriptome analysis of rice endosperm at 6 DAF and 10 DAF also found genes involved in amino sugar and nucleotide sugar metabolism to be overrepresented among the expressed genes (Gao et al. 2013). In wheat, AL and EN genes related to GO categories *transport*, *definition of cell structure*, *cell differentiation*, *lipid metabolic process* and *nitrogen compound metabolic process* are also differentially expressed in these two tissues, while genes encoding proteins involved in GO categories related to *carbohydrate metabolic processes*, *generation of precursor energy* and *macromolecule biosynthetic process* are highly expressed in EN (Gillies et al. 2012; Pfeifer et al. 2014). This is consistent with our results for AL- and EN-specific DEGs. It has been suggested that Zn, K, Ca and Fe concentrations in grains are much higher in AL than in EN (Iwai et al. 2012; Lu et al. 2013a; Zhao et al. 2014). Our results support this suggestion because genes encoding Zn, Mn, Cu and Fe transporters are more highly expressed in AL compared to EN. Together, our data indicate that both OVT and AL tissues contribute significantly to nutrient transport into the EN during rice seed development. Collectively, they support the model of a conserved molecular machinery controlling seed development and grain filling in monocots (Sreenivasulu and Wobus 2013).

The coordination of hormone synthesis and signalling is important during seed development, but information on the expression of hormone-related genes in seed tissues is rather limited for crops. It has been suggested that auxin is present in high concentrations during all stages of seed development and that IAA provides positional information during AL differentiation in maize seeds (Forestan et al. 2012). CK has a prominent role during early seed development and decreases gradually during seed maturation (Locascio et al. 2014). CK-related genes are more highly expressed in the maternal tissue, especially in the ETC in maize and wheat seeds. Similar to CK, the highest concentration of BR is found during early seed development and gradually decreases during seed maturation (Jiang and Lin 2013). GA concentrations are dynamically regulated during early and late seed development stages, while the concentration of ABA is higher during seed maturation (Seo et al. 2006; Liu et al. 2013). In barley, ABA metabolism is higher in EN and AL at 16 and 25 DAF (Seiler et al. 2011), while CK and GA metabolism is higher at 8 and 16 DAF (Thiel et al. 2012). Our results show that genes related to auxin biosynthesis and signalling had higher expression levels in OVT at 4 and 8 DAF, with a small decrease at 16 DAF. Likewise, the genes involved in BR and CK signalling were more highly expressed in OVT and CC during early developmental stages. The genes related to ABA synthesis and signalling have higher expression in EN and AL during the late developmental stage (16 DAF), which is consistent with the results found in barley (Seiler et al. 2011). These data suggest a key role of OVT in hormone regulation and nutrient transport to the developing grain (Kanno et al. 2010; Locascio et al. 2014). Our data, together with previous reports (Seiler et al. 2011; Locascio et al. 2014), also suggest that the role and regulation of phytohormones is largely conserved during grain filling in monocots, although we also found specific differences during grain filling stages in rice.

Rice genes that are preferentially and highly expressed at 4 and 8 DAF are related primarily to photosynthesis, central metabolism and protein translation, while genes expressed highly at 16 DAF are related to protein, lipid and fatty acid metabolism, and stress response. Similar shifts in metabolic and biochemical activities have been found in proteomics data from developing rice seeds (Lee and Koh 2011), thus establishing a good correlation between the transcriptome and proteome data for expected metabolic changes (Sun et al. 2012). To date, only few genes have been characterized for their roles associated with rice grain development. Among them, *OsMADS29* (*Os02g0170300*) regulates the degradation of the nucellus and nucellar projections in rice and is highly expressed in the nucellus and nucellar projections during early developmental stages (3 to 6 DAF), but its expression is low in EN and further decreases at 8 DAF (Yang et al. 2012; Yin and Xue 2012). *OsGIF1* (*Os04g0413500*) encodes a cell-wall invertase required for carbon partitioning during early grain filling stages (3 to 15 DAP) (Wang et al. 2008), and the protein is localized in the OVT. We found that *OsMADS29* and *OsGIF1* are preferentially expressed in OVT at 4 and 8 DAF, and expressed at a very low level in EN at 4, 8 and 16 DAF.

The high number of genes encoding TFs that are differentially expressed during early grain development indicate the complex regulation of activities in the different seed tissues at this stage compared to the mature grain. We found that the expression of genes for MADS, ARF, HB and ARR-B TFs in OVT and MYB and NAC TFs in CC was overrepresented at 4 and 8 DAF. Most of the motifs revealed by MEME analysis in DEGs at these stages also represent DNA sequences known to be bound by members of the MADS, ABI3VP1 and MYB families, thus establishing a strong correlation with the overrepresented OVT and CC TFs. Furthermore, the co-expression network analysis suggested those TFs (e.g. Os03g0753100, a MADS TF, expressed at OVT 4 DAF and Os07g0129700, a HB TF, expressed at OVT 8 DAF) also tightly associated with other target genes that are enriched in the function of transporters, and primary metabolism. In EN, the overrepresented genes encoding TFs belong to the MYB, MADS, NAC and NF-Y families. The bZIP TF genes were mainly expressed in AL and EN, which is consistent with the suggestion from previous reports that bZIP TFs are expressed in AL and the EN tissues in rice and maize, and bind ABRE motifs to mediate the ABA-induced transcription (Xue et al. 2012; Li et al. 2014). Similarly, in maize endosperm during seed development, overrepresented genes encoding TFs also belong to bZIP, MADS, NAC, MYB and SBP families (Lu et al. 2013b; Zhan et al. 2015). In Arabidopsis, known seed-specific TFs include members of the MADS, ARR-B TFs, and bZIP TF families (Le et al. 2010; Khan et al. 2014a, 2014b). In the Arabidopsis funiculus, which connects the seed with the maternal plant and is the only direct route for nutrient transport into the seed, MYC TFs have been reported to regulate the auxin response (Khan et al. 2015). In barley, bZIP and MYB TFs are abundantly present during the transition from grain maturation to germination; while ARF and AUX/IAA TF family members are preferentially expressed in the embryo of germinating seeds (Sreenivasulu et al. 2008; Thiel et al. 2012; Zhang et al. 2016). These reports together with our results suggest that the above identified TF families are generally important for monocot grain development. Our data also revealed genes for novel TFs whose functions are currently unknown. For example, *OsWRKY109* (*Os05g0129800*) had its highest expression in OVT at 4 DAF, but its biological function and regulatory network remain to be identified. Thus, the differentially expressed rice TF genes provide useful information to further dissect the regulatory network controlling rice grain development.

## Conclusion

Together, our comprehensive analysis of genes that are expressed in different tissues of developing rice grains and the subsets of genes that are differentially regulated in these tissues provides new insights into metabolic activities and regulatory transcription factors involved in the developmental and physiological processes. The information on tissue- and stage-specific genes as well as regulatory proteins is a useful resource for future functional genomics studies of rice grain development and grain filling as well as for the design of novel biofortification strategies.

## Materials and Methods

### Plant Material and Growth Conditions

Rice plants (*Oryza sativa* cv. Nipponbare) were grown in hydroponic solutions at 28 °C. Solutions for the hydroponic system were prepared as previously described (Wang et al. 2013). Rice grains were collected at 04, 08 and 16 days after flowering (DAF), de-husked and immediately frozen by placing the collection tubes in liquid nitrogen. Five sub-regions of the rice grains, including CC, NE, OVT, EN and AL were dissected for further analysis (see below). The time points were selected based on the reported differentiation of the main cell types (Krishnan and Dayanandan 2003). NE is differentiated into a single layer cell at around 4 DAF, while the enlarged cells and thickenings of NE are noticed at 8 DAF (Krishnan and Dayanandan 2003). CC are visible as greenish cell layer from 4 DAF to 16 DAF. OVT locates in the ventral side of the endosperm, in conjunction with NE (Krishnan and Dayanandan 2003). EN cells are gradually developing and enlarging through 4 to 16 DAF, and the outer layer of EN cells differentiate into AL at about 16 DAF.

### Cryostat Settings, Mount Slides Preparation and Laser Capture Micro-Dissection (LCM)

Three grains were used for each sample preparation. Cryostat was set to specific conditions with the knife at − 18 °C and specimen at − 20 °C. Thirty μm of trim and 10 μm of fine trim were used. Two to three rice grains from the same developing stage, i.e. 4, 8 or 16 DAF, were mounted in the mould with optimal cutting temperature compound (O.C.T. compound). The mounted samples were incubated at − 20 °C for 5 min. The fixed samples were then removed from the mould for further dissection. One drop of 70% ethanol was added onto the membrane slide. The slide was put upside down and was soaked and warmed in 70% ethanol solution. The slide was attached to the membrane surface of the microscope and was immediately placed back to the cryostat, further drying was allowed for 20 min and the samples were processed using the LCM. PALM LMC settings were adjusted to 65–75% of energy and focus. After Köhler-Illumination, the lens was changed to 10x or 20x objective. The laser-cut tissues were catapulted into an eppendorf cap and then the eppendorf tube was unmounted from the microscope. 350 μl RLT Buffer from Qiagen RNeasy Micro Kit was added to the cap and tube, vortexed for 5 min and incubated at room temperature (RT) for 5 min.

### RNA Extraction, Pre-Amplification and RNA Sequencing

The RNA extraction was done with the Qiagen RNeasy Micro Kit following manufacturer’s instruction manual. All RNA samples were adjusted to a concentration of 100 pg/μl prior to pre-amplification. Pre-amplification for the Illumina sequencing was done using the Clonetech SMARTer Ultra Low kit following the user’s manual with small modifications. In brief, the RNA samples were subjected to first strand cDNA synthesis, followed by 15 cycles of Long Distance (LD) PCR. The PCR amplified cDNA was subsequently purified by immobilizaiton on AMPure XP beads for 15 min at RT, resuspended in 15 μl nuclease-free water and stored at − 20 °C for further experiments and library preparation.

Three biological replicates for each tissue were sequenced at the Functional Genomics Center Zurich (FGCZ). Correlation coefficients of two biological replicates between tissues ranged from 0.85 to 0.9 for most samples, indicating that the results were highly reproducible. Biological replicates of poor quality (i.e., with low correlation coefficients) were excluded, therefore, 28 samples in total were used for detailed analysis [NE_4DAF(3), OVT_4DAF(3), CC_4DAF(2), EN_4DAF(1), NE_8DAF(3), OVT_8DAF(2), CC_8DAF(1), EN_8DAF(2), NE_16DAF(3), OVT_16DAF(1), CC_16DAF(2), EN_16DAF(2), AL_16DAF(3)] (Table S8). The TruSeq SR Cluster Kit v4-cBot-HS (Illumina, Inc., California, USA) was used for cluster generation using 8 pM of pooled normalized libraries on the cBOT. Sequencing were performed on the Illumina HiSeq 2500 single end 126 bp using the TruSeq SBS Kit v4-HS (Illumina, Inc., California, USA).

Threshold for data analysis were chosen to be *p* < 0.001 and RNA-seq reads higher than 10. For a pair-wise comparison, gene expression level change higher than 2-fold and *p*-value< 0.05 were chosen. Reads were aligned with the STAR aligner (Dobin et al. 2012) to the cv. Nipponbare reference genome IRGSP1.0 (Tanaka et al. 2008). Gene expression counts were computed with R/Bioconductor. Differential expression was assessed with the Bioconductor packages edgeR (Robinson et al. 2009). Genes were considered as differentially expressed if the p-value was below 0.01 and the expression fold-change was greater than 2.

### qRT-PCR Validation

Reverse transcription was performed using Superscript-III Reverse Transcriptase (Invitrogen, Carlsbad, USA). The diluted cDNA samples were used as templates for RT-PCR and real-time PCR. The internal control genes used for RT- PCR were rice *UBIQUITIN 5* (*OsUBQ5*) and *ELONGATION FACTOR 1-ALPHA* (*OsEF-1α*). Real-time PCR was performed using SYBR Premix Ex Taq (Takara, Dalian, China) on a Rotor-Gene 3000 (Corbett Research, QIAGEN, Hilden, Germany) detection system and software, according to the manufacturer’s instructions. The primers used for qPCR validation are provided in Supplementary Table 7.

### Data Analysis

The enriched GO categories of subregion or stage-specific DEGs were analyzed in PlantGSEA (Yi et al. 2013) (The Plant GeneSet Enrichment Analysis, http://structuralbiology.cau.edu.cn/PlantGSEA/). Fisher test and cut-off for FDR-adjusted p-value < 0.01 was used to screen the GO term enrichment. The significantly changed metabolic pathway analysis was carried out using the KEGG (Tanabe and Kanehisa 2012) server (Kyoto Encyclopedia of Genes and Genomes, http://www.genome.jp/kegg/) with the threshold of p-value < 0.01. The functional gene set was annotated by TIGR (Rice Genome Annotation, http://rice.plantbiology.msu.edu/index.shtml) and RiceDB (http://ricedb.plantenergy.uwa.edu.au/). The categories of transcription factors were downloaded from PlantTFDB (http://planttfdb.cbi.pku.edu.cn/) for further analysis. DEGs were also assigned and visualized using MapMan v3.6. Default parameters were used and the background of mapping was TIGR7 rice protein and IPR Interpro.

Motif analysis was conducted using the MEME (Multiple EM for motif elicitation, http://meme-suite.org/) Suite analyzing tools (Bailey et al. 2009). 1000 bp proximal or distal to the translational start site ATG of the identified DEGs was extracted from RAPDB (http://rapdb.dna.affrc.go.jp/). Over-represented cis-motifs of a width of 6–10 nucleotides were selected. The enriched known motifs were analyzed in AME (McLeay and Bailey 2010) (Analysis of Motif Enrichment) tools by JASPAR plants database (http://jaspar.genereg.net) with the threshold of p-value < 0.05.

Gene co-expression analysis was performed as described previously (Coman et al. 2014). In brief, the Pearson correlation coefficients between DEGs were calculated. The analysis was visualized in Cytoscape (Cline et al. 2007).

The data discussed in this publication have been deposited in NCBI’s Gene Expression Omnibus and are accessible through GEO accession number GSE153954.

## Supplementary information

**Additional file 1: Figure S1**. Representative images of rice grain thin sections before and after LCM. Representative light microscope images of NE, CC OVT and AL at 4, 8 and 16 days after flowering (DAF). Serial LCM of tissues was performed on thin sections of grains 16 DAF. Before, before LCM; After, after LCM; NE: Nucellar Epidermis; CC: Cross Cells; OVT: Ovular Vascular Trace; EN: Endosperm; AL: Aleurone Layer.

**Additional file 2: Figure S2**. Summary of differentially expressed genes (DEGs) and principle component analysis (PCA) for each rice grain tissue and stage during development. a Correlation of NE, CC, OVT, EN and AL at 4, 8, and 16 days after flowering (DAF) with different biological replicates. b PCA of rice grain tissues. Principle components in one to three collectively represent 79% of the variance in the dataset. NE: Nucellar Epidermis; CC: Cross Cells; OVT: Ovular Vascular Trace; EN: Endosperm; AL: Aleurone Layer.

**Additional file 3: Figure S3.** qRT-PCR validation of tissue-specific genes. Top three genes from each tissue were chosen from the list of DEGs. Values are the average of three technical replicates. Data from two biological replicates are shown. The error bars are from three technical replicates. The gene annotations are listed. NA represent novel genes without annotation. The expression was normalized to the expression of rice *UBIQUITIN 5* (*OsUBQ5*) gene.

**Additional file 4: Figure S4.** Gene Ontology (GO) analysis for the molecular functions of the DEGs in the developing rice grains. Significantly (*p* < 0.01) overrepresented GO terms in (a) OVT at 4, 8 and 16 DAF and (b) AL as compared to CC, NE, OVT and EN are shown as heatmaps.

**Additional file 5: Figure S5.** Co-expression analysis for OVT and CC specific genes in different stages of rice grain development. Co-expression datasets of a OVT- and b CC-specific genes were analysed and a graphic views produced using Cytoscape. Blue: OVT_04, yellow: OVT_08, red: OVT_16, green: bridge TF, and light blue: central TF

**Additional file 6: Figure S6.** Expression profiles of DEGs related to hormone metabolism and transporters in rice grain tissues during development. a Genes related to gibberellin (GA) biosynthesis, signaling and transporters. b Genes related to ethylene (ET) metabolism, transporters and receptors c Genes related to amino acid transport. d Genes involved in nitrate (N), sulfate (S) and phosphate (P) transport. The gene-normalized signal intensities are shown in the heat maps using Z-Scores. DAF: days after flowering.

**Additional file 7: Table S1.** Kyoto Encyclopedia of Genes and Genomes (KEGG) analysis for OVT, CC and EN. The overrepresented pathways are shown by *p*-values < 0.05.

**Additional file 8: Table S2.** KEGG analysis for the AL. The overrepresented pathways are shown by p-values < 0.05.

**Additional file 9: Table S3.** Differentially expressed TF genes and their expression level in each tissue and grain development stages.

**Additional file 10: Table S4.** Expression level of hormone related genes in each tissue and grain development stages.

**Additional file 11: Table S5.** Expression level of transporter genes in each tissue and grain development stages.

**Additional file 12: Table S6.** Expression level of transporter families in each tissue and grain development stages.

**Additional file 13: Table S7**. qPCR primers used in this study.

**Additional file 14: Table S8.** Number of total counts and uniquely mapping rate in each sample.

## Data Availability

All data generated or analyzed in this study are included in this article and the supplementary information files. Further, the data discussed in the publication have been deposited in NCBI's GEO and are accessible through GEO accession number GSE153954.

## Abbreviations

LCM: Laser capture microdissection
CC: Cross cells
NE: Nucellar epidermis
OVT: Ovular vascular trace
EN: Endosperm
AL: Aleurone layer
DEGs: Differentially expressed genes
