# Supplementary figures and images for: Genome Wide Analysis of the Transcriptional Profiles in Different Regions of the Developing Rice Grains
Source: Rice (N Y). 2020 Sep 7;13:62. doi: 10.1186/s12284-020-00421-4 (PMC7477059; doi:10.1186/s12284-020-00421-4)

**Fig. S2**


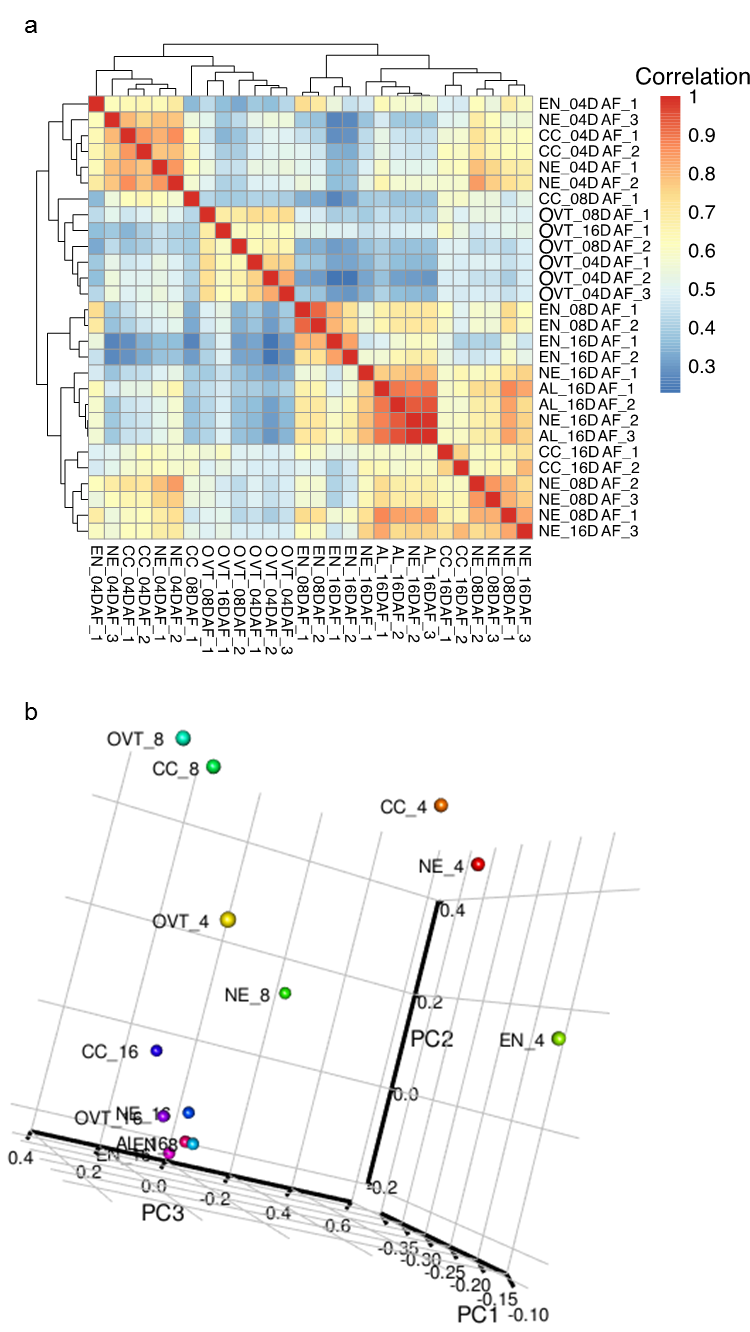

Supplement: Supplementary file 2 — Additional file 2: Figure S2. Summary of differentially expressed genes (DEGs) and principle component analysis (PCA) for each rice grain tissue and stage during development. a Correlation of NE, CC, OVT, EN and AL at 4, 8, and 16 days after flowering (DAF) with different biological replicates. b PCA of rice grain tissues. Principle components in one to three collectively represent 79% of the variance in the dataset. NE: Nucellar Epidermis; CC: Cross Cells; OVT: Ovular Vascular Trace; EN: Endosperm; AL: Aleurone Layer. [file 12284_2020_421_MOESM2_ESM.docx]

**Fig. S3**


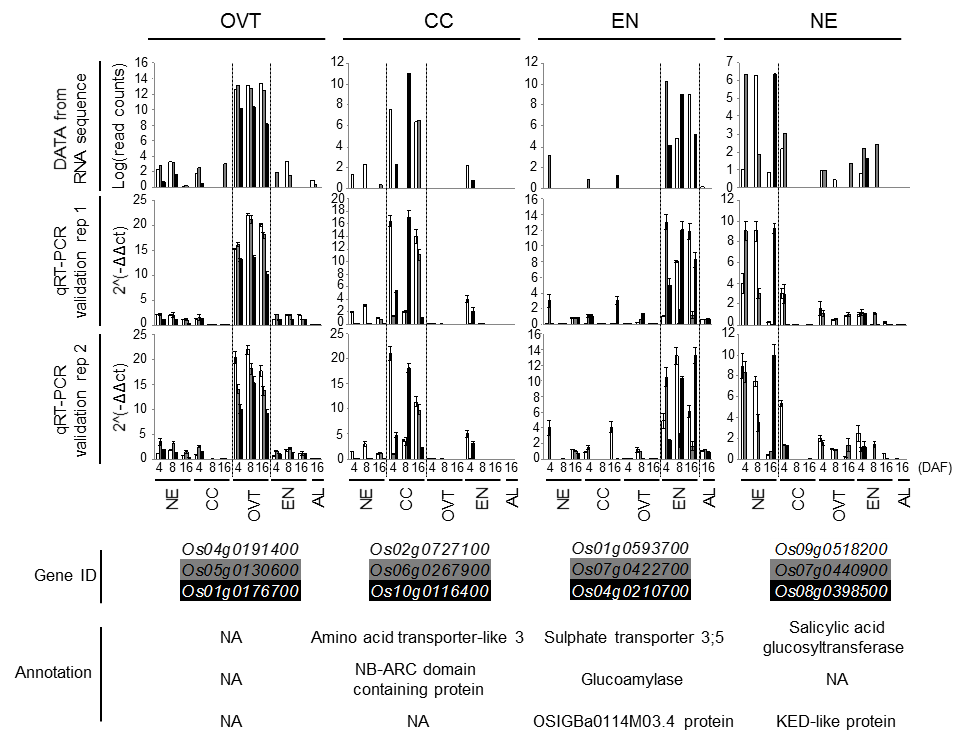

Supplement: Supplementary file 3 — Additional file 3: Figure S3. qRT-PCR validation of tissue-specific genes. Top three genes from each tissue were chosen from the list of DEGs. Values are the average of three technical replicates. Data from two biological replicates are shown. The error bars are from three technical replicates. The gene annotations are listed. NA represent novel genes without annotation. The expression was normalized to the expression of rice UBIQUITIN 5 (OsUBQ5) gene. [file 12284_2020_421_MOESM3_ESM.docx]

**Fig. S4**


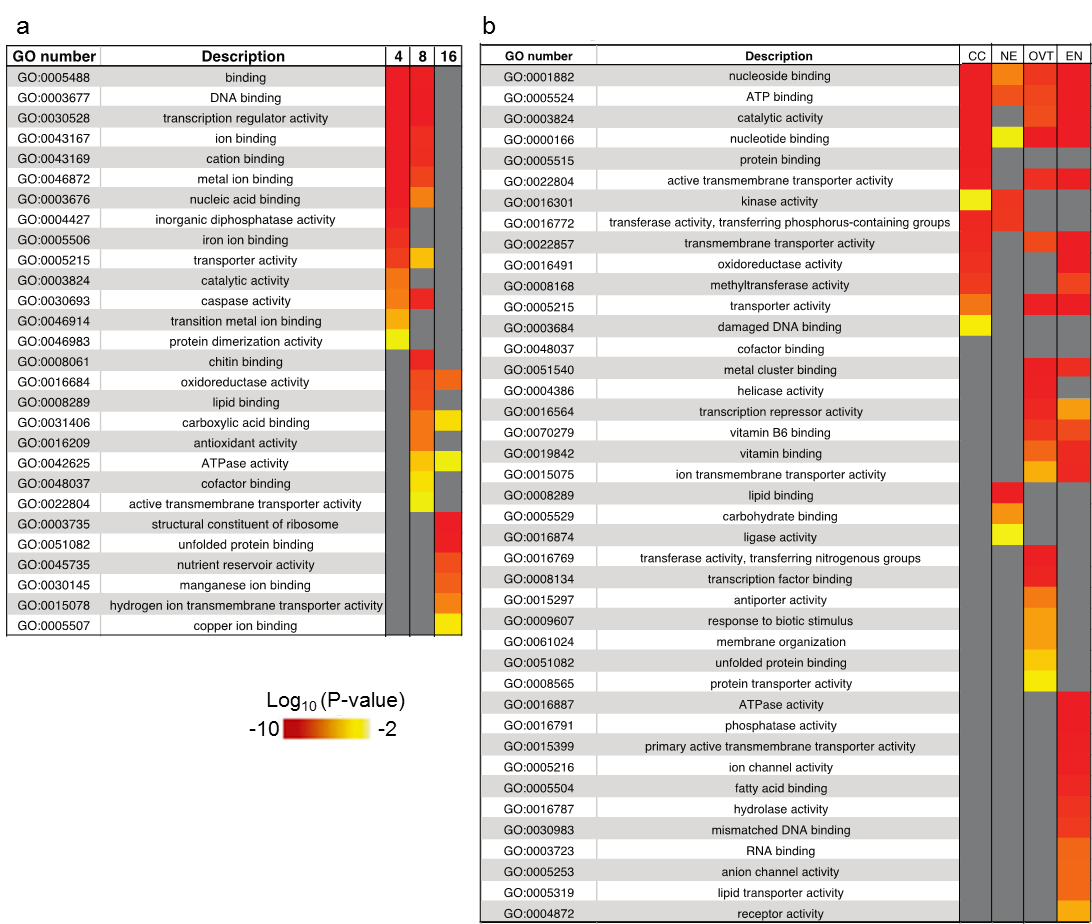

Supplement: Supplementary file 4 — Additional file 4: Figure S4. Gene Ontology (GO) analysis for the molecular functions of the DEGs in the developing rice grains. Significantly (p < 0.01) overrepresented GO terms in (a) OVT at 4, 8 and 16 DAF and (b) AL as compared to CC, NE, OVT and EN are shown as heatmaps. [file 12284_2020_421_MOESM4_ESM.docx]

**Fig. S5**


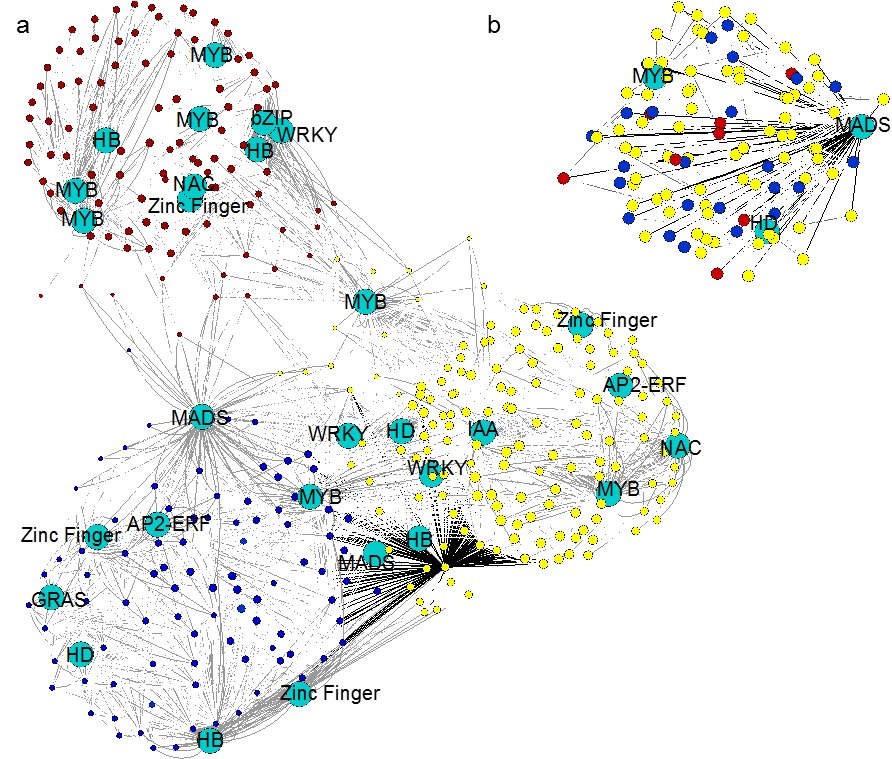

Supplement: Supplementary file 5 — Additional file 5: Figure S5. Co-expression analysis for OVT and CC specific genes in different stages of rice grain development. Co-expression datasets of a OVT- and b CC-specific genes were analysed and a graphic views produced using Cytoscape. Blue: OVT_04, yellow: OVT_08, red: OVT_16, green: bridge TF, and light blue: central TF [file 12284_2020_421_MOESM5_ESM.docx]

**Fig. S6**

**
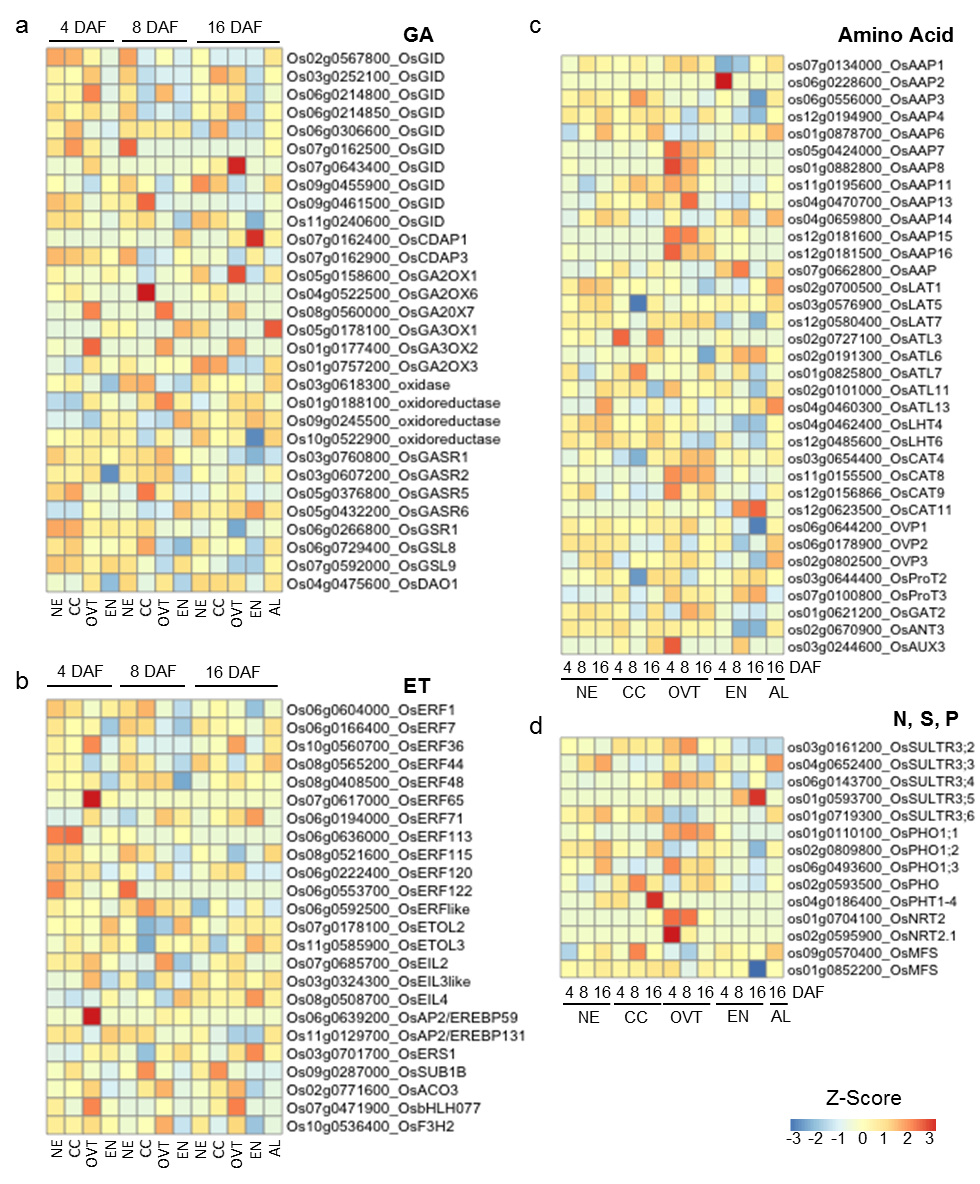
**

Supplement: Supplementary file 6 — Additional file 6: Figure S6. Expression profiles of DEGs related to hormone metabolism and transporters in rice grain tissues during development. a Genes related to gibberellin (GA) biosynthesis, signaling and transporters. b Genes related to ethylene (ET) metabolism, transporters and receptors c Genes related to amino acid transport. d Genes involved in nitrate (N), sulfate (S) and phosphate (P) transport. The gene-normalized signal intensities are shown in the heat maps using Z-Scores. DAF: days after flowering. [file 12284_2020_421_MOESM6_ESM.docx]
