# Supplementary material for: Genome Wide Analysis of the Transcriptional Profiles in Different Regions of the Developing Rice Grains
Source: Rice (N Y). 2020 Sep 7;13:62. doi: 10.1186/s12284-020-00421-4 (PMC7477059; doi:10.1186/s12284-020-00421-4)
Supplement: Supplementary file 7 — Additional file 7: Table S1. Kyoto Encyclopedia of Genes and Genomes (KEGG) analysis for OVT, CC and EN. The overrepresented pathways are shown by p-values < 0.05. [file 12284_2020_421_MOESM7_ESM.docx]

**Table S1. Kyoto Encyclopedia of Genes and Genomes (KEGG) analysis for OVT, CC and EN.**

The over-represented pathways are shown by p-values <0.05

| **KEGG pathway** | **No.** | **p-value** |
| --- | --- | --- |
| **OVT** | | |
| Biosynthesis of secondary metabolites | osa01110 | 4.16E-05 |
| Metabolic pathways | osa01100 | 2.78E-05 |
| Amino sugar and nucleotide sugar metabolism | osa00520 | 3.29E-05 |
| Glycolysis / Gluconeogenesis | osa00010 | 6.70E-05 |
| Starch and sucrose metabolism | osa00500 | 7.61E-05 |
| **CC** | | |
| Phenylalanine metabolism | osa00360 | 1.09E-03 |
| Phenylpropanoid biosynthesis | osa00940 | 1.94E-03 |
| Plant hormone signal transduction | osa04075 | 3.51E-03 |
| Biosynthesis of secondary metabolites | osa01110 | 9.62E-02 |
| Ribosome | osa03010 | 6.28E-31 |
| Oxidative phosphorylation | osa00190 | 1.09E-04 |
| Protein processing in endoplasmic reticulum | osa04141 | 2.23E-03 |
| Pentose phosphate pathway | osa00030 | 8.80E-03 |
| Glycerophospholipid metabolism | osa00564 | 1.86E-02 |
| Endocytosis | osa04144 | 1.95E-02 |
| Metabolic pathways | osa01100 | 3.10E-02 |
| Spliceosome | osa03040 | 4.59E-02 |
| **EN** | | |
| Photosynthesis | osa00195 | 1.91E-14 |
| Metabolic pathways | osa01100 | 5.74E-07 |
| Glycerophospholipid metabolism | osa00564 | 1.38E-02 |
| Biosynthesis of secondary metabolites | osa01110 | 3.75E-02 |
| Porphyrin and chlorophyll metabolism | osa00860 | 4.00E-02 |
| Ascorbate and aldarate metabolism | osa00053 | 1.32E-03 |
| Carbon fixation in photosynthetic organisms | osa00710 | 5.32E-03 |
