# Supplementary material for: Genome Wide Analysis of the Transcriptional Profiles in Different Regions of the Developing Rice Grains
Source: Rice (N Y). 2020 Sep 7;13:62. doi: 10.1186/s12284-020-00421-4 (PMC7477059; doi:10.1186/s12284-020-00421-4)
Supplement: Supplementary file 8 — Additional file 8: Table S2. KEGG analysis for the AL. The overrepresented pathways are shown by p-values < 0.05. [file 12284_2020_421_MOESM8_ESM.docx]

**Table S2. KEGG analysis for the AL.**

The overrepresented pathways are shown by p-values <0.05

| **KEGG pathway** | **No.** | **p-value** |
| --- | --- | --- |
| Biosynthesis of secondary metabolites | osa01110 | 3.93E-05 |
| Metabolic pathways | osa01100 | 5.59E-05 |
| Glycolysis / Gluconeogenesis | osa00010 | 2.70E-03 |
| Ubiquinone biosynthesis | osa00130 | 2.75E-03 |
| Carbon fixation in photosynthetic organisms | osa00710 | 4.59E-03 |
| Protein processing in endoplasmic reticulum | osa04141 | 5.95E-03 |
| RNA degradation | osa03018 | 9.37E-03 |
| Phosphatidylinositol signaling system | osa04070 | 1.14E-02 |
| Pyruvate metabolism | osa00620 | 1.37E-02 |
| Glutathione metabolism | osa00480 | 2.62E-02 |
| Fatty acid biosynthesis | osa00061 | 4.21E-03 |
| Steroid biosynthesis | osa00100 | 1.09E-02 |
| Carotenoid biosynthesis | osa00906 | 1.42E-02 |
| Glycerolipid metabolism | osa00561 | 1.78E-02 |
| RNA transport | osa03013 | 3.51E-02 |
| Mismatch repair | osa03430 | 8.10E-06 |
| DNA replication | osa03030 | 1.75E-05 |
| Ascorbate and aldarate metabolism | osa00053 | 2.15E-03 |
| Amino sugar and nucleotide sugar metabolism | osa00520 | 2.33E-03 |
| Oxidative phosphorylation | osa00190 | 2.09E-02 |
| N-Glycan biosynthesis | osa00510 | 2.14E-02 |
| Ribosome biogenesis in eukaryotes | osa03008 | 2.26E-02 |
| Plant-pathogen interaction | osa04626 | 2.59E-02 |
| Phenylpropanoid biosynthesis | osa00940 | 3.35E-02 |
| Sulfur metabolism | osa00920 | 3.39E-02 |
| Plant hormone signal transduction | osa04075 | 4.59E-02 |
