# Supplementary material for: Genome Wide Analysis of the Transcriptional Profiles in Different Regions of the Developing Rice Grains
Source: Rice (N Y). 2020 Sep 7;13:62. doi: 10.1186/s12284-020-00421-4 (PMC7477059; doi:10.1186/s12284-020-00421-4)
Supplement: Supplementary file 13 — Additional file 13: Table S7. qPCR primers used in this study. [file 12284_2020_421_MOESM13_ESM.docx]

**Table S7. qPCR primers used in this study**

| **GeneID** | **Primer Name** | **Direction** | **Primer sequence (5'-3')** |
| --- | --- | --- | --- |
| Os04g0191400 | O1 | F | CTGTCACTGGAACAGCCTCA |
|  |  | R | GTTCATCTGGCTGTTGTGGA |
| Os05g0130600 | O2 | F | GGCGAGCTCACTTCTTGTTC |
|  |  | R | ACTTGGGCGCCTTTGGTT |
| Os01g0176700 | O3 | F | CACGTCTCGAGGAAGAGAGG |
|  |  | R | CCGATGTTCTTCACCGGTAT |
| Os02g0727100 | C1 | F | GACCTGGAGCAGAGTTGGAG |
|  |  | R | GAGCAGAGGAAGTCGTGGAG |
| Os06g0267900 | C2 | F | AATACACGTCGGCCTTATCG |
|  |  | R | TGCACGTTGTAATGGCAA AT |
| Os10g0116400 | C3 | F | ATTCTTGGTGGTTCGACTGG |
|  |  | R | GGACTTGATGGATGGCAAGT |
| Os09g0518200 | N1 | F | CAACGACAGAGGGGTTGAGT |
|  |  | R | CCATGAACTCCTTGCTCCTC |
| Os07g0440900 | N2 | F | GCAAGAAGGAATCCCAACTG |
|  |  | R | GGTGGTCATCTCACGGATCT |
| Os08g0398500 | N3 | F | AGCCAAGGAACATCACAAGG |
|  |  | R | CCTTCTTCTCCTTGCCCTTC |
| Os01g0593700 | E1 | F | TGCTTCCTCATCTTCCTCGT |
|  |  | R | ATATCGAGCTCGGGTTGATG |
| Os07g0422700 | E2 | F | AACCAAGGACGCTTTCAAGA |
|  |  | R | CGTAAGTTCGGCTCCTTCTG |
| Os04g0210700 | E3 | F | GACCATAATGTTCCGTGAAGG |
|  |  | R | TCTGGAAACCCCTCAAATGT |
| Os03g0234200 | UBQ5 | F | ACCACTTCGACCGCCACTACT |
|  |  | R | ACGCCTAAGCCTGCTGGTT |
